# Drug Discovery for Chagas Disease: Impact of Different Host Cell Lines on Assay Performance and Hit Compound Selection

**DOI:** 10.3390/tropicalmed4020082

**Published:** 2019-05-17

**Authors:** Caio Haddad Franco, Laura Maria Alcântara, Eric Chatelain, Lucio Freitas-Junior, Carolina Borsoi Moraes

**Affiliations:** 1 Brazilian Biosciences National Laboratory, National Centre for Research in Energy and Materials, Campinas, SP 13083-970, Brazil; caiohaddadfranco@gmail.com (C.H.F.); lauramalcantara@outlook.com (L.M.A.); luciofreitas@usp.br (L.F.-J.); 2 Department of Microbiology, Institute of Biomedical Sciences, University of São Paulo, São Paulo, SP 05508-000, Brazil; 3 Drugs for Neglected Diseases Initiative, 1202 Geneva, Switzerland; echatelain@dndi.org

**Keywords:** *Trypanosoma cruzi*, high content screening, phenotypic screening, chagas disease drug discovery, host cells, host-parasite interactions

## Abstract

Cell-based screening has become the major compound interrogation strategy in Chagas disease drug discovery. Several different cell lines have been deployed as host cells in screening assays. However, host cell characteristics and host-parasite interactions may play an important role when assessing anti-*T. cruzi* compound activity, ultimately impacting on hit discovery. To verify this hypothesis, four distinct mammalian cell lines (U2OS, THP-1, Vero and L6) were used as *T. cruzi* host cells in High Content Screening assays. Rates of infection varied greatly between different host cells. Susceptibility to benznidazole also varied, depending on the host cell and parasite strain. A library of 1,280 compounds was screened against the four different cell lines infected with *T. cruzi*, resulting in the selection of a total of 82 distinct compounds as hits. From these, only two hits were common to all four cell lines assays (2.4%) and 51 were exclusively selected from a single assay (62.2%). Infected U2OS cells were the most sensitive assay, as 55 compounds in total were identified as hits; infected THP-1 yielded the lowest hit rates, with only 16 hit compounds. Of the selected hits, compound FPL64176 presented selective anti-*T. cruzi* activity and could serve as a starting point for the discovery of new anti-chagasic drugs.

## 1. Introduction

Chagas disease, caused by the protozoan *Trypanosoma cruzi*, is one of the most significant human parasitic infections in Latin America, affecting approximately 7 million people [1]. This insect-borne disease, which can also be transmitted congenitally and through blood transfusion and organ transplantation [2] causes more than 5000 deaths annually in the Brazilian territory alone [3] and a recent report suggests this mortality rate might be underestimated [4]. Current treatment for Chagas disease is limited to two drugs—benznidazole (BZL) and nifurtimox (NFX)—which not only have several contraindications and side effects but also have variable cure rates when administered to chronic stage patients [5,6,7,8,9]. Recent clinical candidates for Chagas disease include posaconazole and ravuconazole—two CYP51 inhibitors that were repurposed from antifungal chemotherapy. However, clinical failure due to low efficacy against Chagas disease when used in monotherapy [10,11] has highlighted the need for continued drug discovery efforts to find new chemotypes and especially compounds with novel mechanisms of action against *T. cruzi* [12].

In the mammalian host, *T. cruzi* exists in two morphologically and functionally different stages—the invasive and non-replicative stage, the trypomastigote and the replicative stage; the intracellular amastigote. Recently, a subpopulation of non-replicative amastigotes has been implicated in drug tolerance and treatment evasion [13,14]. Cell-based assays for compound screening against the replicative intracellular amastigote and high content screening (HCS) assays in particular, have become the gold standard for discovery of new anti-infective drugs for Chagas disease as they enable screening against the parasite stage involved with disease pathogenesis, even in the absence of validated drug targets [15,16]. Cell based assays with *T. cruzi* are facilitated by the fact that this parasite is able to invade, differentiate and multiply in virtually all nucleated mammalian cells [17]. Thus, several HCS protocols have been developed using a variety of mammalian cell lines as host cells, such as bovine embryo skeletal muscle (BESM), human hepatoma Huh-7 [18], mouse fibroblast 3T3 [19,20,21], human osteosarcoma U2OS [22], mouse myoblast C2C12 [21], rat myoblast H9c2 [23] and monkey kidney epithelial Vero [24]. *T. cruzi* is a complex organism with high phenotypic variability between strains in terms of drug susceptibility [25,26,27,28] and postulated tissue tropism [29,30,31] which can hinder comparison of results from different in vitro and in vivo assays, complicating drug discovery efforts. Host cells and tissues add another layer of complexity: the invasive and replicative capacity of *T. cruzi* can vary depending on the host cells [30] and some studies suggest that host cell metabolism regulates *T. cruzi* metabolism and replicative capacity [32]. It can also be argued that differential compound susceptibility depending on the host cell or tissue type might also influence the course of the therapeutic outcome in vivo and indeed it is not known whether *T. cruzi* has a variable response to drug treatment in different infected tissues in vivo. 

Considering these points, it can be argued that distinct host cells might impact compound activity on *T. cruzi* differently. However, a systematic exploration of how host cells interfere with *T. cruzi* susceptibility to compounds in vitro has so far been lacking. To address this matter, we have evaluated the impact of different cell lines used as host cells for *T. cruzi* infection in a standardized HCS assay in the context of small molecule compound library screening. 

## 2. Materials and Methods 

Compounds: The LOPAC^®1280^ (Library Of Pharmacologically Active Compounds) small scale library was purchased from Sigma-Aldrich, benznidazole was kindly donated by Nortec Química and nifurtimox was provided by Epichem Pty. FPL64176 was resourced from Sigma-Aldrich.

Cells: Monkey kidney epithelial cells LLC-MK2 (*Macaca mulatta*) and VERO (*Cercopithecus aethiops*) were kindly provided by S. Schenkman (Federal University of São Paulo, Brazil) [33] and A. Tanuri (Federal University of Rio de Janeiro, Brazil) [34]. The human cell lines U2OS (osteosarcoma, BCRJ0304) and THP-1 (monocytic leukemia, BCRJ0234) were obtained from the Rio de Janeiro Cell Bank (BCRJ, Brazil), whereas the rat (*Rattus norvegicus*) skeletal myoblast L6 cell line (IFO50364) was obtained from the Japanese Collection of Research Bioresources (JCRB, Japan). The *T. cruzi* Y strain was also originally provided by S. Schenkman and a clone (Y-H10) was generated through limiting dilution [35] at the Institut Pasteur Korea (South Korea); Sylvio X10/1 strain clone was donated by M. Miles (London School of Hygiene and Tropical Medicine, UK); CL Brener strain was donated by J. F. Silveira (Federal University of São Paulo). All mammalian cells and *T. cruzi* strains were cultured in RPMI 1640 medium (Sigma-Aldrich), supplemented with 20% (*v/v*) fetal bovine serum (FBS, Gibco) and an antibiotic solution of 100 U/mL penicillin and 100 µg/mL streptomycin (Gibco), under an atmosphere containing 5% CO_2_ at 37 °C. Mammalian cell lines were passaged every 3 to 4 days, with a maximum of 20 passages for adherent host cells and 10 for THP-1. *T. cruzi* strains were maintained in tissue culture of LLC-MK2 cells, as described [28], for a maximum of 7 passages. Cell cultures were routinely inspected for mycoplasma contamination using the MycoAlert detection kit (Lonza). 

**Figure 1:**
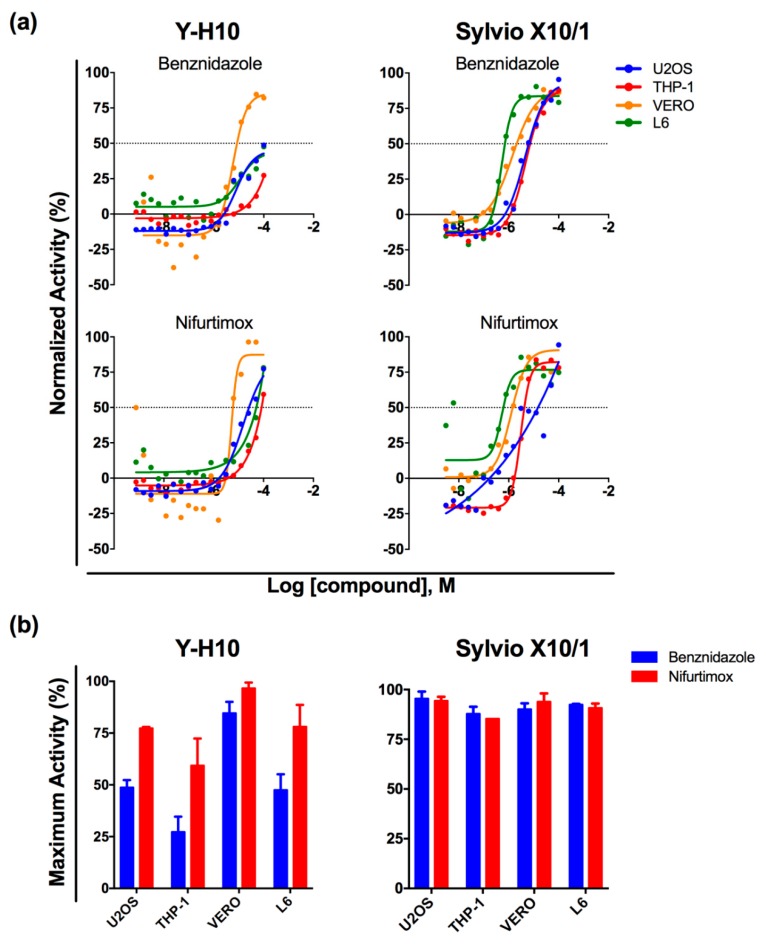
**Activity profile for reference drugs benznidazole and nifurtimox against *T. cruzi* Y-H10 and Sylvio X10/1 infecting different host cells.** (**a**) Dose-response curves for reference compounds. X-axis: compounds normalized activity (in %), Y-axis: log of compound concentration (in Molar units). Spots refer to mean values of antiparasitic activity for either Y-H10 (left) and Sylvio X10/1 (right) infecting U2OS (blue), THP-1 (red), Vero (orange) and L6 (green) from two independent experiments. (**b**) Maximum observed activity (Y-axis) for nifurtimox (red) and benznidazole (blue) in each host cell assay (X-axis) for *T. cruzi* Y-H10 (left) and Sylvio X10/1 (right). Data represent the means and standard deviation from two independent experiments.

Reference compound solutions and library preparation: benznidazole and nifurtimox were dissolved in dimethyl sulfoxide (DMSO, Sigma-Aldrich) to prepare stock solutions of 20 mM and 10 mM, respectively. Aliquots of stock solutions were kept frozen at below −80 °C, protected from light and submitted to a maximum of three cycles of freezing-thawing. Dose-response curves were prepared as described [28], with starting test concentrations of either 400 µM (data shown in Table 2) or 100 µM (data shown in Figure 1) for benznidazole and 100 µM for nifurtimox. The LOPAC library was acquired in a 96-well-microplate format, dissolved in DMSO at a stock concentration of 10 mM and manually formatted into 384-well polystyrene stock microplates (Grener BioOne). A second set of 1 mM stock plates was prepared by transferring 2 µL of 10 mM compound solution into 18 µL DMSO. Stock plates were sealed and stored at −20 °C.

HCS assay and compound screening: The assay was based on the methodology previously reported [36], with some modifications. On the first day of the assay, cells were counted in suspension and seeded onto 384-well cell culture microplates (µClear, Greiner BioOne) at 300 (L6), 600 (Vero), 700 (U2OS) and 7000 (THP-1) cells/well, in 40 µL RPMI 1640 complete medium (L6, Vero and U2OS) and incubated for 24 h. THP-1 was plated at 25 µL RPMI 1640 complete medium containing 50 ng/mL phorbol 12-myristate 13-acetate (PMA, Sigma-Aldrich) and incubated for 48 h. After the incubation period, trypomastigotes harvested from the supernatant of *T. cruzi*-infected LLC-MK2 cultures were added onto assay plates at a multiplicity of infection (MOI) of 4 trypomastigotes:1 plated host cell for THP-1 and 20 trypomastigotes:1 plated host cell for L6, Vero and U2OS. The final volume in all wells was adjusted to 50 µL/well. After a further 24 h of incubation, compounds from the 1 mM stock plates were added to plates to the final concentration of 10 µM, 1% DMSO (*v/v*), in a volume of 60 µL/well. Wells containing non-infected cells and infected cells treated with 1% DMSO (*v/v*) were used as positive and negative controls, respectively. After compound addition, plates were incubated for 96 h under the controlled conditions mentioned above. The LOPAC library was screened once for each cell line assay and each run included two plates containing dose-response curves of benznidazole and nifurtimox and one plate containing infected cells treated with 1% DMSO (*v/v*) in all wells as controls. At assay endpoint (after 96 h of compound exposure), plates were fixed for 15 min with a solution of 4% paraformaldehyde (Sigma-Aldrich) in phosphate buffered saline (PBS, Sigma-Aldrich) pH 7.4, followed by two steps of washing with PBS and staining with 5 µM Draq5 (in PBS) solution for 20 min. Confirmatory screenings were performed in dose-response following the protocol described above, using cherry-picked (selected 11 hits, screened in duplicate in the U2OS assay and in singlet in the L6 assay) or fresh re-supplied compound FPL64176 (screened in triplicate against Y-H10, Sylvio X10/1 and CL Brener strains).

Image acquisition and processing: for assay development and primary screening with the Y-H10 clone, four images of each well were acquired using the high content system Operetta (PerkinElmer) with a 20x magnification lens (long working distance), 90% excitation and an exposure time of 800 ms. A far-red filter was selected for wavelengths at 620 nm–640 nm excitation/650 nm–760 nm emission. After acquisition, images were submitted to high content analysis using the Harmony^®^ software (PerkinElmer) for primary screening and phenotypic characterization and drug assays on cells infected with Y-H10. Sylvio X10/1 experiments and hit confirmation assays were performed using the IN Cell 2200 high content system for image acquisition and Investigator software for image analysis (GE), using similar settings as described above. The analysis identified, segmented and counted the individual nuclei and cytoplasm of host cells, as well as detected and counted the parasites present in the host cell cytoplasmic region. The analysis building blocks were customized for each infected cell line assay, yielding measurements of the total number of cells, total number of infected cells, total number of intracellular parasites (spots) in the images acquired. The mean number of parasites/infected cell was calculated by dividing the number of intracellular parasites (spots) by the number of infected cells. 

Data analysis: data were analyzed as described [28]. Briefly, the ratio between the number of infected cells and the total number of cells detected by the software was defined here as the infection ratio (IR). IR values were normalized to negative and positive controls in order to determine normalized antiparasitic activity. The cell ratio (CR) was defined as the ratio between the total number of cells in a well and the average number of cells per well for the intraplate negative control and was used as prediction of cytotoxicity against the host cell. The Z’-factor was calculated as described [37]. Screening data were processed using Excel (Microsoft) and Spotfire (Tibco) and data for dose-response curves were plotted using Prism 7.0a (GraphPad) to generate sigmoid curves by non-linear regression of the values and determination of EC_50_ and CC_50_ data by interpolation. Maximum activity value (MA, in %) refers to the highest mean value of antiparasitic activity measured for each drug. EC_50_ was defined as the effective compound concentration value, corresponding to 50% of normalized activity after 96 h of compound incubation; similarly, the CC_50_ value was determined as the compound concentration that reduced the number of cells by 50% of the average number of cells in the intraplate negative control wells. The ratio between the CC_50_ value and the EC_50_ value was used to determine the selectivity index (SI) of a compound. When EC_50_ or CC_50_ values could not be determined, the value was estimated as lower than the minimum concentration tested (EC_50_) or higher than the maximum concentration tested (CC_50_). A hierarchical cluster analysis of compounds was performed using online tools available at http://chemminetools.ucr.edu/.

## 3. Results

### 3.1. T. cruzi High-Content Assay Development for Different Host Cell Lines

In an attempt to address the impact of the host cell on *T. cruzi* response to compounds, a previously developed HCS assay [28] was adapted to use four cell lines: U2OS, Vero, L6 and THP-1. Different cell plating densities were tested for each cell line (data not shown) to obtain the ideal confluency for high content imaging after 144 h of assay. Concomitantly, initial assay development and optimization established that the same trypomastigote to host cell ratio or multiplicity of infection (MOI), could be used for the U2OS, Vero and L6 cells (data not shown). THP-1 cells, however, required a much lower MOI of 4, as higher MOIs led to an almost complete host cell lysis at assay endpoint (data not shown). The multiparametric analysis resulting from infection with the H10 clone of the Y strain of *T. cruzi* under these conditions can be seen in Table 1. The optimization of cell plating densities was carried out in order to have most of the well surface covered at sub-confluency within 144 h of assay (and thus facilitate image analysis) and accordingly variation in the final density of cells was observed, with an average of approximately 2900 cells in the Vero assay, 1,900 cells in the L6 assay and 1,200 cells in the U2OS assay. The THP-1 assay was associated with the lowest cell densities, with an average of only 320 cells imaged per surface area. A typical image of each infected host cell system at assay endpoint, after standardization, is shown in Figure S1. 

Despite the final cell density of Vero and L6 assays being somewhat similar and considering that they were infected under the same MOI, the average infection ratio (IR) for Vero cells (28%) was less than half that calculated for L6 (77%). U2OS cells displayed an intermediate ratio of infection (52%) and approximately 45% less cells per surface area than Vero and L6, which was associated with U2OS slower population doubling time and reduced parasite-driven cell lysis at the assay endpoint (data not shown). THP-1 displayed high infection rates (73%) and the lowest cell number per surface area imaged. Infection patterns become even more markedly distinct when the MOI is considered, as THP-1 cells were infected with a MOI 5 times lower than the other cell types but resulted in very high infection ratio. Another remarkable difference was observed for the mean number of intracellular parasites/infected cells, as the output for the THP-1 assay (15) was 3-fold the number measured in the Vero assay (5). While the L6 assay resulted in the highest infection ratio, the mean number of intracellular parasites/cell was of intermediate value (7), when compared to other cell types. Similar results are observed for the total number of intracellular parasites in the well, however, the THP-1 number is reduced as a consequence of lower cell density compared to other cell lines (Table 1). However, when the same assays were performed with a different *T. cruzi* strain, Sylvio X10/1, the resulting infection profile was markedly different. While THP-1 cells were also heavily infected by this strain (infection ratio of 77%), the L6 cells had the lowest infection ratio (31%) and the U2OS and Vero were more heavily infected by the Sylvio X10/1 than the Y-H10 strain (Table S1). 

The response to antichagasic drugs was then investigated. The dose-response relationships for benznidazole and nifurtimox were determined in the four *T. cruzi*-infected cell line assays. The Y-H10 clone presented natural tolerance to benznidazole and to a much lower extent, nifurtimox [35]. The response of this clone to benznidazole varied greatly between different host cells, with a remarkably low response in the THP-1 host cell (Figure 1). The maximum activity detected for benznidazole against the Y-H10 clone was more heterogeneous, depending on the host cell used, ranging from ca. 30% in THP-1 to ca. 80% in Vero cells. The same pattern of heterogeneity was observed in response to nifurtimox, although the overall efficacy was higher (above 50% in all assays). In contrast, the response of Sylvio X10/1 to both drugs was more homogeneous and comparable in efficacy for the same host cell type. With the exception of THP-1, which proved to be the host cell associated with decreased efficacy for both strains and both drugs (but to a much lower degree in the case of Sylvio X10/1), no association was observed between parasitic load (number of total parasites, Table 1 and Table S1) and/or host cell type and drug efficacy (Figure 1).

### 3.2. Assay Performance in Compound Library Screening

**Figure 2:**
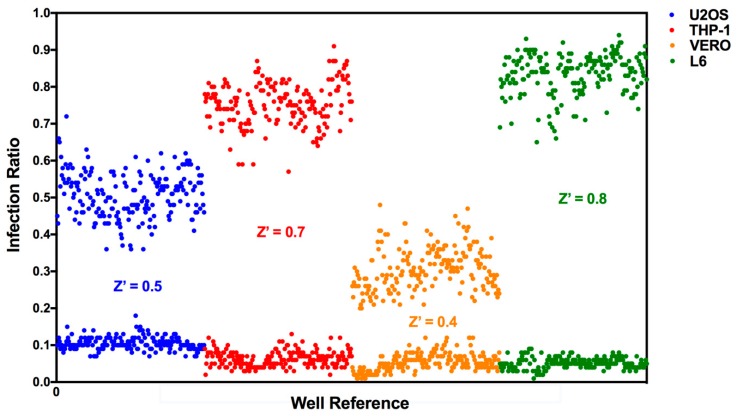
**Screening assay performance depends on the host cell.** Z’-factors and distribution of negative (DMSO-treated) and positive (non-infected) controls for LOPAC^1280^ library screening using different host cell lines infected with *T. cruzi* Y-H10: U2OS (blue dots), THP-1 (red), Vero (orange) and L6 (green). X-axis: infection ratio values for each control well, Y-axis: well reference for assay plates. Data were plotted from controls of six assay plates (four library compound plates + two dose-response control plates).

A commercial library containing 1280 pharmacologically-active compounds was screened against *T. cruzi* Y-H10 infecting the four cell lines: U2OS, THP-1, Vero and L6. All microplates tested presented acceptable Z’-factor values, which varied according to the host cell assay (Figure 2). The lowest average Z’-factor (0.4 ± 0.07) was seen in the Vero host cell screen, followed by the U2OS screen (0.5 ± 0.02), THP-1 (0.7 ± 0.03) and finally the L6 screen (0.8 ± 0.04). Additionally, the variation within each control set, here shown by the Y-axis dispersion of the spots, also influenced the Z’-factor calculation; in general, different host cell line assays showed minimum variation in detected infection ratios (false positives/cell debris and inclusions identified as amastigotes) for positive controls (non-infected cells) and a more heterogeneous variation in the negative control (mock-treated infected cells) infection ratio values, which reached a variation of ca. 25% (Vero) to ca. 40% (U2OS and THP-1, data not shown).

**Figure 3:**
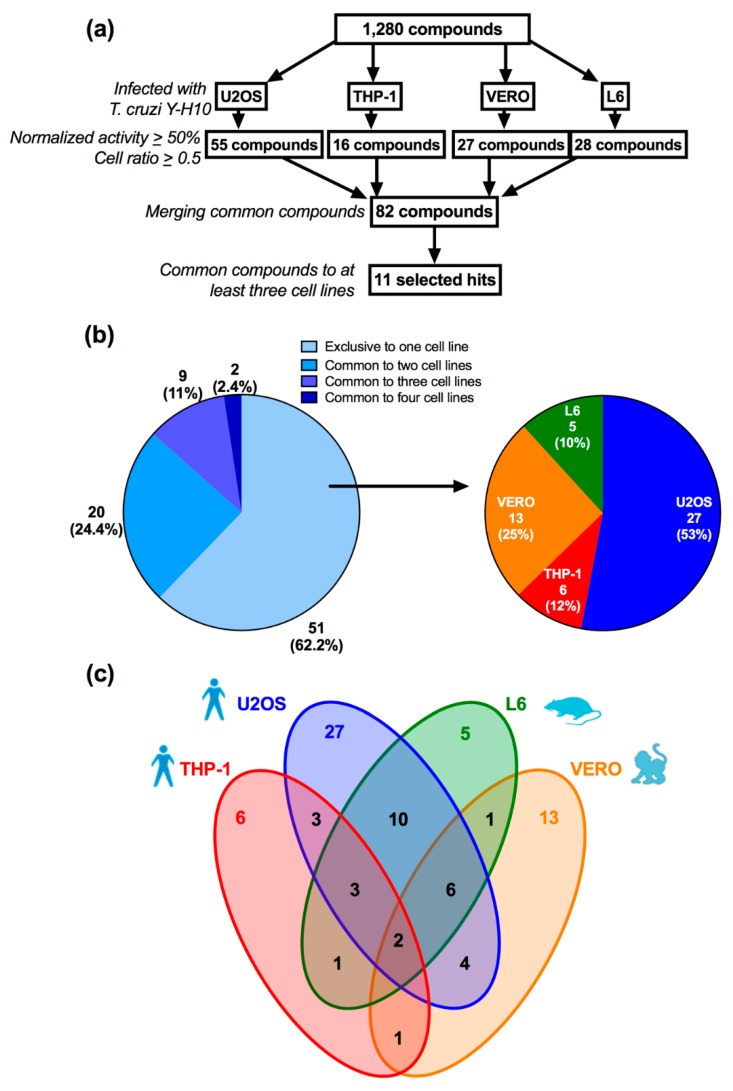
**LOPAC^1280^ compound library screening scheme and hit selection.** (**a**) All 1,280 compounds were screened against each cell line infected with *T. cruzi* Y-H10 strain: U2OS, THP-1, VERO and L6. Different numbers of potential hit compounds were selected for each host cell assay according to the following criteria: normalized activity superior to 50% and cell ratio higher than 0.5, totaling 82 compounds, of which only 11 were common to at least three cell line screens (selected hits). (**b**) Left: percentage of the selected hits yielded by one (exclusive), two, three or four cell line screens. Right: distribution of the exclusive selected hits by specific cell line. (**c**) Diagram shows distribution and sharing of potential compounds among cell lines: colored numbers indicate the number of exclusive active compounds for each cell line screen, while numbers in the intersection regions show shared potential compounds for the respective cell line. U2OS (blue), THP-1 (red), VERO (orange) and L6 (green).

The screening assay was performed according to the scheme shown in Figure 3A. All 1280 compounds were tested against the distinct cell line assays at a single concentration of 10 µM. Compounds were selected based on normalized antiparasitic activity (NA) > 50% and cell ratio (CR) > 0.5. This first prioritization filter yielded a different number of potential compounds for each cell line assay: 55 compounds for U2OS, 16 compounds for THP-1, 27 compounds for Vero and 28 compounds for L6. Disregarding the duplicates, there were 82 unique compounds; of these, only two (2.4%) were a hit common to all four screens, whereas nine (11%) and 20 (24.4%) were selected compounds common to three and two host cell line screens, respectively. Interestingly, 51 compounds (62.2%) were selected exclusively from one assay only (Figure 3B—left chart). Further examination of the compounds selected from a single host cell screen showed that infected U2OS cells yielded the highest percentage of hits, with 27 exclusive compounds selected (53%), followed by Vero with 13 compounds (25% and with THP-1 and L6 yielding the lowest numbers, with 6 (12%) and 5 (10%) exclusive compounds, respectively (Figure 3B—right chart—and Figure 3C). For the 82 compounds selected, the infected lines which had the most compounds in common were U2OS and L6 cells (21). The other double, triple or quadruple combinations yielded < 10 shared compounds among the cell lines (Figure 3C).

**Figure 4:**
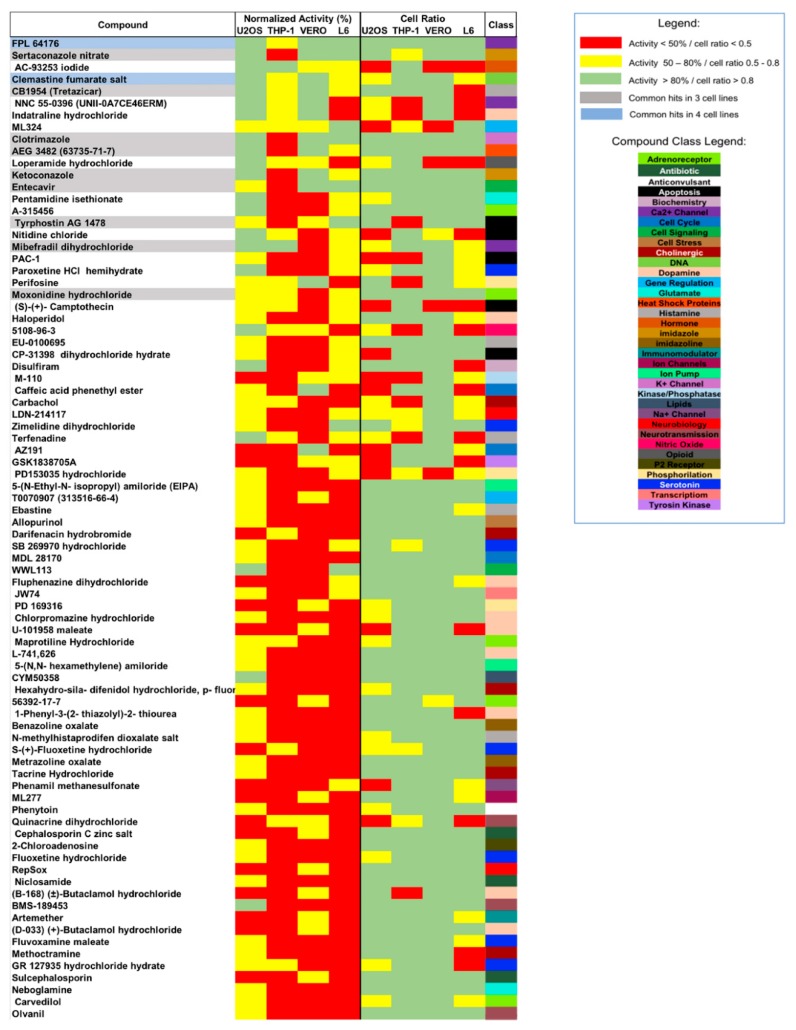
LOPAC ^1280^ library screening against *T. cruzi* infecting distinct cell lines: hit compound profiling. Normalized activity (left columns) and cell ratio values (right columns) for selected compounds assayed at 10 µM against four cell lines infected with *T. cruzi*. Color highlighting indicates the range of activity/cell ratio: < 50%/0.5 (red), 50–80%/0.5–0.8 (yellow) and > 80/0.8 green. Grey highlighting in the compound list shows hit compounds selected in 3 cell line screens while light blue highlighting indicates selected hits common to all four cell line screens. The box on the right indicates the classification of hit compounds (according to library compound profiling) represented by the color code indicated in the legend box.

The activity profiling of the 82 compounds is shown in Figure 4 in descending order of activity against all infected cell lines. The data show that the higher number of hits in the U2OS screen is due to individual higher activity of compound this assay. Regarding host cell cytotoxicity, measured here by cell ratio (CR) values, L6 and U2OS cell lines seemed relatively more sensitive, with 17 compounds resulting in CR < 0.5 in the L6 assay and 14 compounds for the U2OS assay; the Vero cell line was apparently the least cytotoxicity-sensitive cell line, with only 5 compounds resulting in CR < 0.5 while 10 compounds resulted in CR within this range for THP-1 cells. 

A hierarchical agglomerative cluster analysis was performed based on selected compound structures but no direct correlation could be established with the agglomerative cluster results and the activity or cytotoxicity of the compounds, except for a common subgroup projected for hit-selected compounds moxonidine hydrochloride, CB1954 and AEG3482 (Figure S2-A). The selected compounds were distributed into 35 pharmacological categories, marked according to the color pattern in Figure 4. Classes of compounds acting on neurotransmission and the cellular receptors represented the highest percentage of the selected compounds: dopamine, serotonin and adrenoreceptor classes occupied 11%, 8.5% and 6.1% of the 82 selected compound classes respectively; histamine, apoptosis and cholinergic classes each represented 6.1% of the selected compounds, while the other compound classes were represented by less than 4% of the list (Figure S2-B). However, when pharmacological class frequency in the selected compounds list is compared to the composition of the whole library, it is noticeable that neither adrenoreceptor nor cholinergic classes are enriched within the screening results (Figure S2-B). However, the pharmacological class frequency for the selected hits (those compounds active/selective in at least three cell lines screens) was notably increased when compared to the library composition, especially in the case of imidazoles and heat shock protein compound classes, which presented > 100-fold enrichment in the selected hit list (Figure S2-C). Hit compound classes for Ca^2+^ channel, cell signaling and DNA-targeting were also seen with increased frequency, with > 10-fold enrichment in comparison to the whole library (Figure S2-C).

### 3.3. Hit-Compound Activity Confirmation

Compounds that were active in at least three host cell screens, a total of 11 (highlighted in Figure 4), were selected for confirmatory screening in dose-response against *T. cruzi* Y-H10 infecting U2OS and L6 host cells (Tables S2 and S3). Nifurtimox, which was used as a reference drug, displayed high efficacy (approximately 100%, data not shown) and potency (EC_50_ values of 0.5 µM in the U2OS assay and 2.1 µM in the L6 assay). Some of the selected compounds that were tested also presented high potency, with EC_50_ values < 1.0 µM. All compounds displayed similar levels of potency in both screens, with the exception of mibefradil, which was not active in the L6 assay. In terms of cytotoxicity prediction, the reference drug and most of the tested compounds had satisfactory selectivity and 7 of the 11 selected hits did not generate CC_50_ values against the U2OS host cells, while others had selectivity indexes lower than 10. L6 host cells were more robust than U2OS in terms of cytotoxicity and only one hit compound, moxonidine, was cytotoxic for this cell line. 

In addition to the drugs on this hit list that were previously reported to be active against *T. cruzi*, the compound FPL64176 stood out as a promising new molecule for drug discovery for Chagas disease: its activity was confirmed from a fresh solid stock and it was found to be efficacious and selective against three different *T. cruzi* strains infecting the U2OS cell line (Table 2). To the best of our knowledge, this is the first report of FPL64176 as having anti-*T. cruzi* activity.

## 4. Discussion

Given its ability to infect practically any mammalian nucleated cell, *T. cruzi* is considered to be a promiscuous parasite. We screened a commercial library containing 1280 pharmacologically active compounds to assess the influence of host cell on the anti-*T. cruzi* activity of compounds and the potential impact on screening campaigns and Chagas disease drug discovery. A robust high-content screening assay has been developed and used by our group for assessing compound activity against *T. cruzi* Y strain infecting U2OS cell line [22,28,36,38,39]. This methodology does not require genetically-modified host cells or parasites, nor specific antibody labelling and relies on a single-channel DNA staining for imaging of infected cells. Thus, the approach can be applied to virtually any combination of host cell line and parasite strain. We aimed at standardizing the assay conditions, already well-established for the U2OS cell line, to other infected host cells: THP-1 (human), Vero (monkey) and L6 (rat), thus adjusting the assay protocol for these mammalian cells. All cell lines were adjusted to THP-1 basic culture conditions because modifications in THP-1 culture protocol would be more critical to assay performance, thus RPMI culture medium with 20% FBS was utilized for all mammalian host cells. A clone isolated from the Y strain [35] was used to eliminate inherent strain population variability by using a homogeneous parasite population, thus focusing on the differences between the host cell lines. 

Although most assay parameters were standardized for the host cells, intrinsic system variability and particular cell line characteristics, such as distinct doubling time, resulted in different infection and high content analysis outputs. The total cell number at assay endpoint, for instance, varied considerably between the cell lines. The final cell number was also influenced by the cell lysis provoked by trypomastigote release from infected cells, which varied according to both the cell line and the parasite strain and was more noticeable in the infection of Vero and L6 by the Y-H10 strain (data not shown). THP-1 cells had the lowest total cell number while Vero cells yielded at least five times more cells within the same imaged fields. This is likely due to the fact that this monocytic cell can become terminally differentiated into macrophage-like cells upon phorbol stimulus [40], whereas Vero cells divide approximately every 22 to 24 h ([41] and data not shown). Additionally, the THP-1 protocol involves incubating the cells in the assay plate for a longer period (differentiation process, see methods) and the infectivity profile is superior to that observed in Vero cells, both in terms of IR and parasite load (Table 1, Figure S1). This scenario could contribute to reducing the total cell number from THP-1 infected with Y-H10 strain. THP-1 cells were also heavily infected by the Sylvio X10/1 strain. Interestingly, as *T. cruzi* cell entry into differentiated THP-1 is likely to occur both passively (parasite phagocytosis) and actively (trypomastigote cell invasion) and the MOI used for this cell line was 20% of the proportion used for the other cell lines, THP-1 yielded a much more pronounced infection. 

*T. cruzi in vitro* infection is a result of two main variables, invasion capacity and amastigote doubling time. Under the experimental conditions used in this study (which were set to lead to a saturated infection state, to increase the window between negative and positive controls—an important factor in screening robustness) and at assay endpoint (120 h of infection), it is very likely that more than one infection cycle had occurred. Thus, the contribution of individual factors, invasive capacity and replication doubling times—which seem to vary according to the pair of *T. cruzi* strain and host cell—both contribute to determining the infection outcomes. L6, along with THP-1, presented a superior ratio of infected cells but yielded a lower number of intracellular amastigotes/infected cells for Y-H10 infection, when compared to other lines (Table 1, Figure S1). *T. cruzi* Y strain is classified as highly macrophagotropic rather than myotropic [29,42,43]; therefore, the in vitro invasion of a macrophage-like cell by this strain may be favored in comparison to the other cell lines. Interestingly, Sylvio X10/1 promoted lower infection of L6 cells, with rates almost three times lower than observed for Y-H10 infected L6 cells (Table S1); probably the divergence in a myoblast cell line (L6) infectivity profile between the strains could be associated with strain-specific “tropism,” which might as well be the result of a combination of the variables mentioned above [43]. 

A direct consequence of a superior ratio of infection, if consistent throughout the assay plate, is the generation of a high standard Z’-factor, indicating good quality screening controls and dependable results. L6, THP-1 and U2OS cell line screens yielded excellent Z’-factors, whilst the Vero cell screen exhibited a Z’-factor of moderate quality (Table 1, Figure 2). This lower Z’-factor may be attributed not only to the relatively low infection observed but also to the variability of infection within the control assay plate (Figure S2). Although the main high throughput assay quality control parameter is Z’-factor > 0.5 (of good quality), marginal quality assays (0 < Z’-factor < 0.5) are usually accepted in the case of highly-complex phenotypic experiments [44]. 

Reference compounds exhibited distinct activity profiles in the different host cell lines infected with Y-H10. Benznidazole presented varied efficacy, with highest activity in the Vero cell line, which had the lowest infection ratio with the Y-H10 (and antiparasitic activity was based on the reduction of infection). This was expected as this clone has been associated with tolerance to benznidazole [35]. The lowest level of efficacy was observed in THP-1 cells infected with Y H-10. A different profile was observed for Sylvio X10/1, against which benznidazole presented a more homogeneous and efficacious response in all cell lines tested. Sylvio X10/1 has already been described as susceptible to benznidazole [45]. Despite the varying levels of efficacy observed, it seems that potency correlated with levels of infection and more drug was needed to reach similar levels of efficacy in more heavily infected cells, as seen in Figure 1A for THP-1 for both Y-H10 and Sylvio X10/1, L6 for Y-H10 and U2OS for Sylvio X10/1. Thus, these data suggest that cell lines (which might be associated with varied infection levels due to invasion capacity and amastigote doubling time) and parasite strain (which might have varying levels of tolerance to the drug) both contribute to benznidazole efficacy. The antiparasitic activity of nifurtimox followed a more homogeneous pattern against these two strains, as at least 50% activity was reached in all cell lines infected with both *T. cruzi* strains (Figure 1). 

Amastigotes of infected THP-1 cells were more tolerant to both benznidazole and nifurtimox, when compared to the other cell lines, especially in the Y-H10 strain. In addition to the fact that these host cells were associated with greater infection, the lower activity might be associated with the inherent metabolic status of the cell and hostile cytoplasmic environment of a professional phagocyte (i.e., the PMA-induced differentiated THP-1 cell), the cellular physiology of which might directly influence the progression of parasite invasion, replication and infectivity. This particular cellular status could also interfere with the compounds’ pharmacodynamic properties, thus reducing their antiparasitic efficacy. Drug activity that is variable depending on the macrophage origin has also been reported for another trypanosomatidic intracellular parasite, *Leishmania donovani* [46]. 

The fact that benznidazole tolerance was more pronounced in the Y-H10 strain in THP-1 in comparison to other host cells also suggests that combinations of host cell-parasite interactions might exacerbate tolerance to benznidazole. Therefore, the present data also supports the notion that the infected tissues targeted by the parasites (and their particular infected cells) may also play a role in the variable efficacy of compounds at the intracellular level but further experiments with primary cells and in vivo should be performed to test this hypothesis.

A similar trend was observed in the primary screening, with the infected THP-1 cell screen yielding the lowest number of selected compounds, most probably due to the cell line particularities discussed above. Although extensively used as a human macrophage cell model, including for *Leishmania* infection, this is to our knowledge the first reported use of THP-1 cells for anti-*T. cruzi* compound screening. While Vero and L6 screens yielded intermediate numbers of selected compounds, the U2OS screen resulted in a vast number of active compounds. Additionally, the U2OS screen also had the highest number of the most active compounds (21 compounds with NA > 80%). A genome-wide RNAi screening study has already reported a proportionately higher number of potential host cell targets, which interfere directly with *T. cruzi* infection, within the infected U2OS cell, when compared to infected HeLa cells [47]. Therefore, the higher number of compounds yielded in infected U2OS cells is likely to be due to a higher probability of hitting host cell targets, making the system relatively more promiscuous to compound selection. Alternatively, some of active compounds selected against *T. cruzi*-infected U2OS host cells could require higher concentrations to achieve similar effect on the other infected host cell assays, as the outcome of the activity may also be influenced by the particular compound chemistry (host cell permeability to the compound and the compound mechanism of action). In the activity confirmatory screening, most compounds were active at similar potencies in both U2OS and L6 assays (albeit slightly more potent in the former), with the exception of mibefradil, which did not generate EC_50_ in the L6-infected cells—which could be expected as this compound presented moderate activity already in the primary screening-. Further experiments are needed to address the impact of host cell and its relation to the mechanism of action of compounds but the low number of selected hit compounds common to all cell line screens (2.4% of selected compounds) and the high number of compounds exclusive to a single host cell (62.2% of selected compounds) suggest that primary screening results generated from distinct *T. cruzi*-infected cell lines are highly dependent upon the host cell of choice and its particular interaction with the parasite, thus promoting a host-cell-parasite focused selection and lower than expected data overlap with other screening assay. Nonetheless, as the primary screening campaign was performed (as it is usual) by testing the compound library in a single concentration once in each cell line assay, further experiments involving duplicate screening and hit confirmation in dose-response against different infected host cells would be needed to further explore the impact of host cell in the outcome of screening campaigns and hit selection.

Although there was a high frequency of selected hit compounds from pharmacological classes related to neurotransmission and the central nervous system (CNS), there is no library enrichment associated with these classes of compounds. Also, neurotransmission and CNS classes have already been reported as promiscuous compound classes with frequent off-target-driven hit molecules [24,48]. Greater library enrichment was noticed for imidazole compounds, as expected, but this was also the case for compounds targeting immunomodulatory and heat shock protein pathways to a similar degree. When compound selection was narrowed down to those active against at least three infected cell lines screens (a total of 11 compounds), an enrichment was observed for imidazole and DNA-targeting classes, as well as heat shock protein, Ca^2+^ channel and cell signaling-targeting compounds, indicating that these target classes could be further explored for antichagasic drug discovery. 

Considering the evident differences in compound activity observed in the infected cell lines screen shown in this study, it is likely that the variability observed in this in vitro model is biologically relevant and once the parasite invades different cells and tissues in the mammalian host, compound activity might be locally different. A potential anti-*T.cruzi* hit compound is expected to be active in the majority of the cell lines tested, which suggests that the compound would be selected within the ample in vitro drug assays available. Although there is no guarantee that these compounds would advance in follow-up experiments, it indicates that the target probably occurs in the parasite, independently of a mammalian cell enzyme or structure. 

Among the selected hits tested in dose-response, FPL64176—a benzoylpyrrole compound which acts as a Ca^2+^ activator [49]—was highly active against all *T. cruzi* strains tested. Although disruption of Ca^2+^ homeostasis has been proposed as an approach to Chagas disease treatment [50], with compounds disrupting Ca^2+^ homeostasis described as having anti-*T. cruzi* activity, such as amiodarone [51] and mibefradil [52], FPL64176 was described here for the first time as an anti-*T. cruzi* agent with broad spectrum and selective activity against different infected cell lines and parasite strains. FPL64176 was approximately 10-fold more potent than benznidazole against Y-H10 clone, which is associated with benznidazole tolerance and FPL64176 also presented superior efficacy (higher maximum activity) when compared to the reference drug, thus indicating that it acts through a mechanism that is different from benznidazole and could be used in combination with this compound.

While there may be not a single cell line that is more appropriate or recommendable for *T. cruzi* drug screening, our results point to the need for better understanding of how *T. cruzi* infection in different host cells and tissues might influence response to drugs and, ultimately, treatment outcome. Further experiments could involve the comparison of compound activity in primary cells that are relevant for *T. cruzi* infection in vivo, such as cardiomyocytes and primary macrophages. 

## Figures and Tables

**Table 1:** General phenotypic parameters yielded by *T. cruzi* Y-H10 infection with different host cell lines.

Cell Line	MOI *	Infection Ratio (%)	Number of Host Cells	Intracellular Parasites/Infected Cell	Number of Intracellular Parasites
**U2OS**	20	52 ± 0.1	1241 ± 124	11 ± 0.4	6493 ± 48
**VERO**	20	28 ± 4	2952 ± 101	5 ± 1	3943 ± 402
**L6**	20	77 ± 7	1926 ± 846	7 ± 1	10,708 ±3846
**THP-1**	4	73 ± 4	320 ± 41	15 ± 1	3644 ± 893

* MOI: multiplicity of infection (ratio of trypomastigote to host cell plated). Values indicate mean ± standard deviation from two independent experiments.

**Table 2:** FPL64176 against Y-H10, Sylvio X10/1 and CL Brener *T. cruzi* strains infecting U2OS host cells.

Compound	*T. cruzi* Strains								
	Y-H10			Sylvio X10/1			CL Brener		
	EC_50_ (µM)	S.I.	MA (%)	EC_50_ (µM)	S.I.	MA (%)	EC_50_ (µM)	S.I.	MA (%)
**Benznidazole**	23.3 ± 6.8	>8.6	85.9	3.4 ± 1.5	>70	98.4	4.0 ± 0.3	>208	107.6
**FPL64176**	2.3 ± 0.2	57.7	99.8	3.0 ± 1.6	35.4	97.6	2.2 ± 0.6	>90	111.5

S.I., selectivity index; MA, maximum activity (in %). Values indicate mean ± standard deviation from three independent experiments.

## Supplementary Materials

The following are available online at https://www.mdpi.com/2414-6366/4/2/82/s1, Figure S1: Representative images of *T. cruzi* Y-H10 infecting different host cells. Table S1: General high-content screening parameters yielded by *T. cruzi* Sylvio X10/1 infection with different host cell lines. Figure S2: Hit compounds clustering and frequency distribution. Table S2: Hit activity confirmation for cherry-picked compounds against *T. cruzi* Y-H10 infecting U2OS cells. Table S3: Hit confirmation for cherry-picked compounds against *T. cruzi* Y-H10 infecting L6 host cell.
