# Supplementary material for: Drug Discovery for Chagas Disease: Impact of Different Host Cell Lines on Assay Performance and Hit Compound Selection
Source: Trop Med Infect Dis. 2019 May 17;4(2):82. doi: 10.3390/tropicalmed4020082 (PMC6630705; doi:10.3390/tropicalmed4020082)
Supplement: Supplementary file 1 [file tropicalmed-04-00082-s001.pdf]

Supplementary Information

Drug discovery for Chagas disease: impact of different host cell lines on assay performance and hit compound selection

Caio Haddad Franco[1,2], Laura Maria Alcântara[1,2], Eric Chatelain[3], Lucio Freitas-Junior[1,2] and Carolina Borsoi Moraes[1,2,*]

[1]Brazilian Biosciences National Laboratory, National Centre for Research in Energy and Materials, Campinas – SP 13083-970 Brazil; caiohaddadfranco@gmail.com; lauramalcantara@outlook.com

[2]Department of Microbiology, Institute of Biomedical Sciences, University of São Paulo, São Paulo – SP 05508-000, Brazil; luciofreitas@usp.br

[3]Drugs for Neglected Diseases Initiative, Geneva 1202, Switzerland; echatelain@dndi.org

*Correspondence: carolinaborsoi@gmail.com or cbmoraes@usp.br; Tel.:+55-11-3091-7343

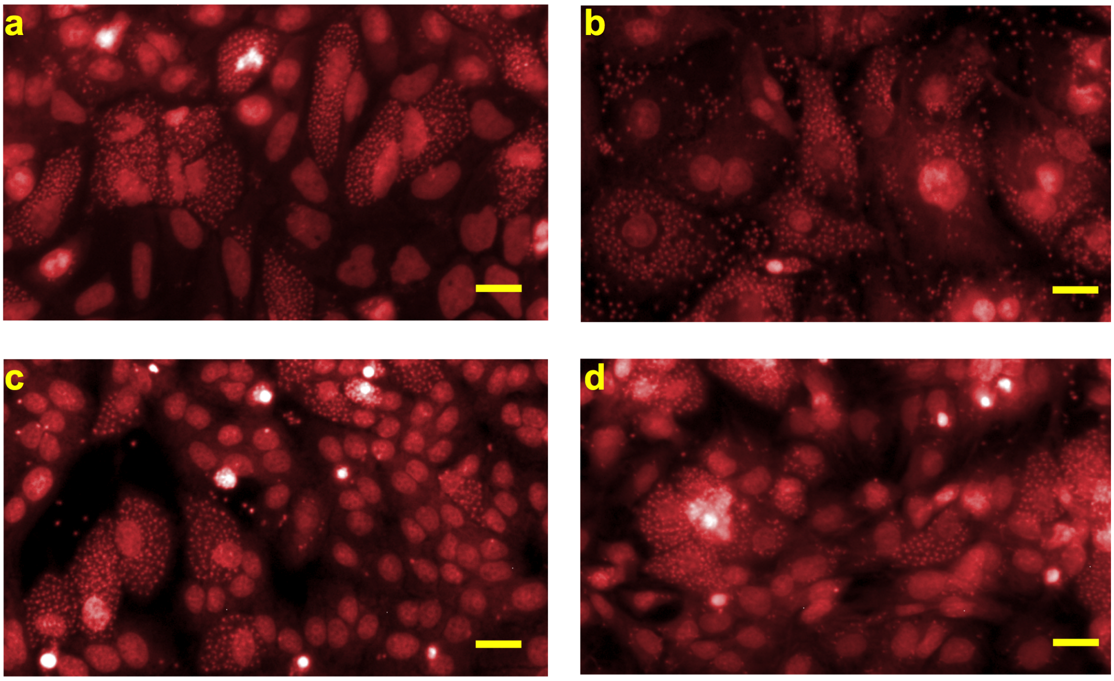

Figure S1. Representative images of *T. cruzi* Y-H10 infecting different host cells. (a) U2OS, **(b)** THP-1, **(c)** Vero and **(d)** L6. Host cell and parasite DNA stained with Draq5 (in red). Bar: 25 µm.

Table S1. General high-content screening parameters yielded by *T. cruzi* Sylvio X10/1 infection with different host cell lines.

Cell line	MOI*	Infection ratio [%]	Number of host cells	Parasite/ infected cell	Number of intracelular parasites
**U2OS**	20	72 ± 10	936 ± 118	17 ± 2	11,478.7 ± 1,155
**VERO**	20	42 ± 1	2,252 ± 29	19 ± 2	17,887.2 ± 2,187
**L6**	20	31 ± 1	1,912 ± 74	24 ± 0.3	13,946.4 ± 536
**THP-1**	4	77 ± 4	1,072 ± 23	13 ± 2	10,937.4 ± 1,476.3

*Multiplicity of infection: ratio of trypomastigote to one host cell in the moment of infection. Values indicate mean ± standard deviation from two independent experiments.

A

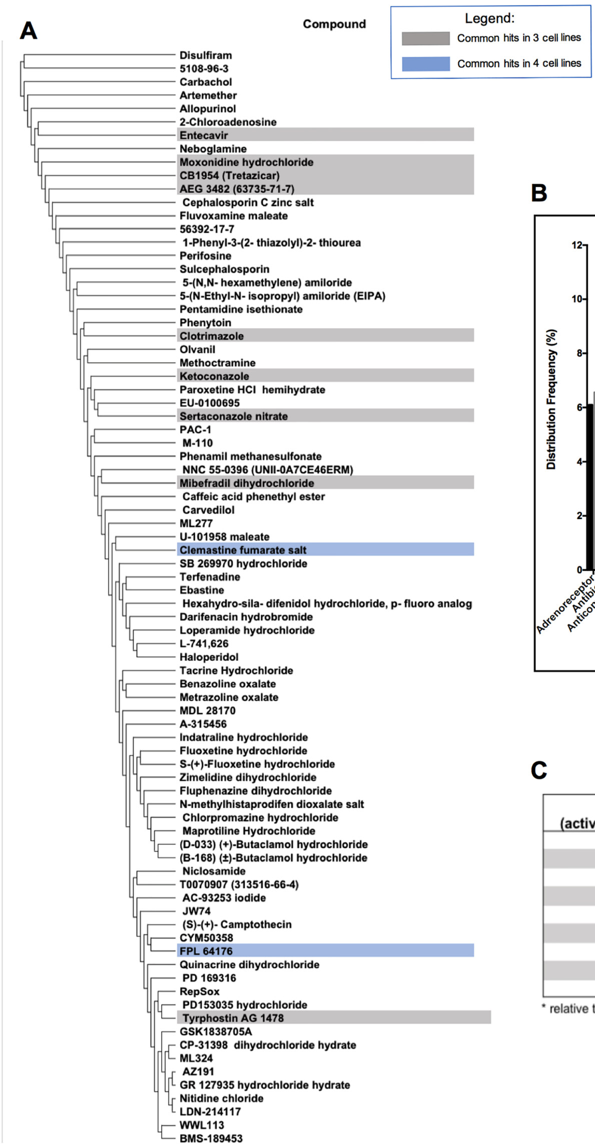

B

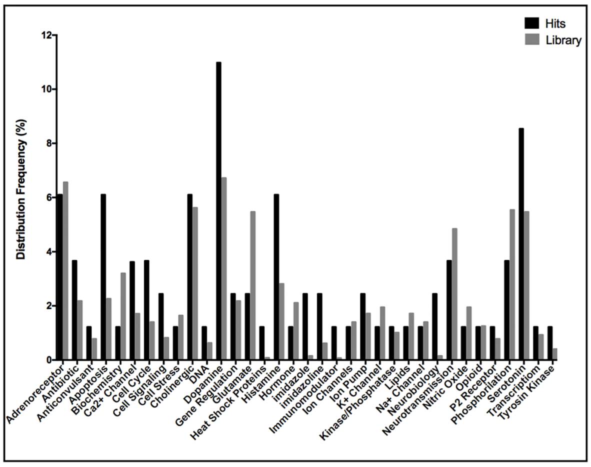

C

Selected hits class (active in at least three cell lineages)	Frequency (%)	Enrichment-fold*
Adrenoreceptor	9.1	1.4
Apoptosis	9.1	4.0
Ca2+ Channel	18.2	10.6
Cell Signaling	9.1	10.6
DNA	9.1	14.4
Heat Shock Proteins	9.1	113.6
Histamine	9.1	3.2
Imidazole	18.2	121.2
K+ Channel	9.1	4.7

* relative to whole library composition frequency

Figure S2. Hit compounds clustering and frequency distribution. a) A single linkage dendogram was built in hierarchical agglomerative clusters based on pairwise compound similarities defined using the Atom Pair descriptors and Tanimoto coefficient (http://chemminetools.ucr.edu). Grey highlights in compound list show *selected hit compounds* in 3 cell lines while light blue highlight common hits in all four cell line screens. Cluster bin cut-off used: 0.4. **b)** Distribution profile comparison between the compound class (X-axis) frequency (in percentage, Y-axis) of the 82 hits selected (black columns) and the frequency in the whole compound library (grey columns**). c)** Compound class of the 11 selected hits which were active in least three cell line screens, their frequency in this selected set and the respective enrichment-fold indicating the increase in frequency ratio in regard to the whole library composition.

Table S2. Hit activity confirmation for cherry-picked compounds against *T. cruzi* Y-H10 infecting U2OS cells.

Compound	*T. cruzi* clone Y-H10		
	EC_{50} (μM)	CC_{50} (μM)	S.I.
**Nifurtimox**	0.5 ± 0.5	25.7*	48.0
**Sertoconazole**	2.2 ± 1.1	ND	> 45
**Ketoconazole**	< 0.2	ND	ND
**CB1954**	< 0.2	ND	ND
**AEG3482**	0.8 ± 0.5	ND	> 128
**Mibefradil**	7.9 ± 2.0	19.5 ± 3.3	2.5
**Clotrimazole**	1.7*	ND	60.6
**Entecavir**	2.2 ± 1.4	ND	> 45
**Moxonidine**	8.1 ± 2.8	20.8 ± 2.3	2.6
**Tyrphostin AG1478**	4.9 ± 1.1	47.7 ± 1.2	9.6
**FPL64176**	2.1 ± 0.2	ND	> 47
**Clemastine**	0.8 ± 0.04	30.5 ± 5.6	33.6

Values show mean ± standard deviation from three independent experiments. *Values obtained in single experiment. ND: the value could not be determined within the concentration range tested.

Table S3. Hit confirmation for cherry-picked compounds against *T. cruzi* Y-H10 infecting L6 host cells.

Compound	*T. cruzi* clone Y-H10		
	EC_{50} (μM)	CC_{50} (μM)	S.I.
**Nifurtimox**	2.1	50.1	24
**Sertaconazole**	1.6	ND	> 62
**Ketoconazole**	< 0.2	ND	-
**CB1954**	NT	NT	-
**AEG3482**	4.2	ND	> 24
**Mibefradil**	ND	-	-
**Clotrimazole**	0.4	ND	> 250
**Entecavir**	2.2	ND	> 46
**Moxonidine**	8.4	14.1	1.7
**Tyrphostin AG1478**	13.9	ND	> 7
**FPL64176**	2.3	ND	> 43
**Clemastine**	1.9	ND	> 52

Values obtained in single independent experiment. ND: the value could not be determined within the concentration range tested. NT: not tested.
